# Predictors of breastfeeding initiation in Hong Kong and Mainland China born mothers

**DOI:** 10.1186/s12884-015-0719-5

**Published:** 2015-11-03

**Authors:** Kris Yuet Wan Lok, Dorothy Li Bai, Marie Tarrant

**Affiliations:** School of Nursing, Li Ka Shing Faculty of Medicine, University of Hong Kong, 21 Sassoon Road, Pokfulam, Hong Kong SAR

**Keywords:** Breastfeeding, Initiation, Chinese, Hong Kong

## Abstract

**Background:**

In recent years there has been a steady influx of immigrants into Hong Kong from Mainland China, where breastfeeding patterns differ. Studies in other regions have found substantial differences in breastfeeding rates between native-born and immigrant mothers. The purpose of this study was to examine factors associated with breastfeeding initiation in Hong Kong born and Mainland China born mothers living in Hong Kong.

**Methods:**

We used a multi-center cross-sectional study design and recruited 2761 new mothers from the postnatal wards of all eight public hospitals in Hong Kong that offer obstetric services. We assessed breastfeeding status as well as various socio-demographic, maternal and birth characteristics. Chi-square tests and multivariable logistic regression were used to identify the predictors of breastfeeding initiation in Hong Kong born and Mainland China born participants.

**Results:**

80.3 % of Hong Kong and 81.1 % of Mainland Chinese born women initiated breastfeeding. In the fully adjusted models, multiparity (Odds Ratio [OR] 0.53, 95 % CI 0.43–0.66) and maternal smoking (OR 0.29, 95 % CI 0.18–0.45) were strongly associated with failure to initiate breastfeeding in both Hong Kong and Mainland China born participants. In Hong Kong born mothers, participants with lower maternal education and those who had a cesarean section were significantly less likely to breastfeed. For Mainland China born mothers, paternal smoking (OR 0.70, 95 % CI 0.49–0.99) and having a pregnancy-related health problem (OR 0.60, 95 % CI 0.38–0.94) were both additional risk factors for not breastfeeding.

**Conclusion:**

This study has identified predictors of breastfeeding initiation in Hong Kong and Mainland China born mothers. Given the current high breastfeeding initiation rates among both groups, antenatal breastfeeding education and promotion programmes need to specifically intervene with sub-groups of pregnant women at risk for not breastfeeding so that their efforts are more strategic and cost-effective.

## Background

The benefits of breastfeeding have been widely recognized in both developing and industrialized countries [1]. International health organizations recommend exclusive breastfeeding for the first six months of life with breastfeeding maintained as an integral part of the infant’s diet for one to two years of age and beyond [1, 2]. Globally, there is an increasing trend of mothers initiating breastfeeding [3], although rates of exclusive breastfeeding remain low and overall duration is short [4–10]. When compared with infant formula feeding, breastfeeding provides unparalleled health benefits to both the infant and the mother. Breastfeeding infants have lower rates of most infectious diseases [11], fewer infectious disease hospitalizations [12–14], and more stable growth trajectories [15]. In the early postpartum period breastfeeding reduces the mother’s risk of postpartum hemorrhage and in the long-term reduces the risk of both breast and ovarian cancers [16, 17].

Over the past 30 years, breastfeeding initiation rates in Hong Kong have increased from 19 % in 1981 [18] to over 85 % in 2012 [19]. Studies in other populations have found several factors associated with breastfeeding initiation, such as older maternal age [20, 21], higher education [10, 22, 23], and higher socioeconomic status [10, 24]. A large-scale population-based Hong Kong birth cohort study conducted in 1997 found similar patterns of breastfeeding initiation, whereby older maternal age and higher education were positively associated with breastfeeding initiation [25]. However, studies of breastfeeding continuation in Hong Kong have found that less educated, lower income and migrant mothers tend to continue breastfeeding for longer periods [26, 27]. Moreover, in recent years, there has been a steady influx of immigrants to Hong Kong from Mainland China [28, 29]. Breastfeeding rates in China have generally been higher than that of Hong Kong [30]. Studies outside of Asia have found that breastfeeding initiation rates differ substantially with ethnicity [31–33]. In the United States (US) Caucasian women initiate breastfeeding at higher rates than Non-Caucasian women [32, 34]. In Hong Kong, there have been few high-quality population-based studies looking at breastfeeding initiation rates and patterns. Furthermore, few studies have compared breastfeeding initiation rates between Hong Kong born and Mainland Chinese born mothers. Although both groups are ethnically Chinese, there are substantial socio-cultural differences between Hong Kong born women and women who have migrated from Mainland China [28]. Given the rapidly changing demographic makeup of the Hong Kong population due to substantial inward migration, it is important to further examine factors associated with breastfeeding initiation between these two groups to better target breastfeeding promotion efforts and to improve breastfeeding outcomes. Thus, the aim of this study was to examine and compare factors associated with breastfeeding initiation in Hong Kong and Mainland China born mothers living in Hong Kong.

## Methods

### Design, setting and participants

Participants were drawn from a territory wide cross-sectional study conducted at all eight public hospitals with in-patient postnatal obstetric services in Hong Kong from April to June 2011. The detailed study methodology is described in detail elsewhere [35]. Briefly, we recruited Chinese-speaking mothers who had just given birth to a live newborn, and who were at least 18 years of age and Hong Kong residents. In Hong Kong, eight public and ten private hospitals offer obstetric services, with public hospitals accounting for over two-thirds of all births to Hong Kong residents [36]. During the postpartum stay, demographic data were collected by maternal self-report using a self-administered questionnaire. A trained research nurse collected the relevant maternal, obstetric, and birth data directly from participants. Breastfeeding initiation was defined as the infant having received human milk at least once during the hospital stay prior to participant recruitment. Breastfeeding status was ascertained from the inpatient log sheet on the postnatal ward and validated with the participants. Study findings are reproted in accordance with the STROBE statement [37].

### Data analysis

Chi-square statistics were used to compare the characteristics of the sample and to assess the maternal and infant characteristics associated with breastfeeding initiation in Hong Kong and Mainland China born women. Multivariable logistic regression analyses were performed to estimate the unadjusted and fully adjusted associations between the study variables and breastfeeding initiation. All variables were entered into the multivariable logistic regression models and odds ratios with 95 % confidence intervals were computed. The Hosmer-Lemeshow test [38] was used to assess the fit of the logistic regression models. The 0.05 level of significance was used throughout the statistical analysis and all data analysis was conducted using Stata version 13.1 statistical software (Stata Corp, College Station, Tx) [39].

### Ethics

Ethical approval for the study was obtained from the Institutional Review Board of the University of Hong Kong/Hospital Authority Hong Kong West Cluster and from all participating hospitals. All participants gave informed written consent.

## Results

Of 3487 postpartum mothers approached, 2863 agreed to participate in the study. Subsequently, 36 participants were excluded because of failure to complete the data collection forms, leaving 2827 participants with complete data (Site A = 221; Site B = 211; Site C = 305; Site D = 308; Site E = 431; Site F = 419; Site G = 544; Site H = 388). Six study hospitals provided us with birth statistics for the study period, from which we have estimated that the recruited participants captured between 50 and 60 % of all eligible pregnant women in the public hospitals during the study period. Among the 2827 participants, 2761 were eligible for this analysis based on their birthplace of either Hong Kong (*n* = 1757) or Mainland China (*n* = 1004). A total of 80.6 % (*n* = 2225) of participants initiated breastfeeding in the hospital, with similar rates among Hong Kong born mothers (80.3 %) and Mainland China born mothers (81.1 %).

The characteristics of the participants are presented in Table 1. Overall, the majority of the participants were aged 30 years or over (64.6 %), married (94.2 %), non-smokers (96.6 %), and without any pre-existing health conditions (91.5 %) or pregnancy-related complications (86.5 %). The majority of births were vaginal deliveries (73.9 %) with full-term (92.2 %) and normal birth weight infants (92 %). When compared with Mainland China born women, participants born in Hong Kong were more likely to be primiparas (59.4 vs. 42.5 %), to have a university degree (27.6 vs. 12.8 %) and higher household income (41.6 vs. 17.8 %), and to work full-time during pregnancy (81.2 vs. 53.8 %) and after giving birth (69.1 vs. 37.9 %).

**Table 1:** Characteristics of the sample

	All subjects	Hong Kong Chinese born	Mainland China born women	*P* value
	(*N* = 2761)	(*N* = 1757)	(*N* =1004)	
Initiated breastfeeding				0.623
Yes	2225 (80.6)	1411 (80.3)	814 (81.1)	
No	536 (19.4)	346 (19.7)	190 (18.9)	
Maternal age (years)				0.001
18–24	265 (9.6)	159 (9.1)	106 (10.6)	
25–29	712 (25.8)	419 (23.9)	293 (29.2)	
30–34	1028 (37.2)	697 (39.7)	331 (33.0)	
≥35	756 (27.4)	482 (27.4)	274 (27.3)	
Parity				<0.001
Primiparous	1470 (53.2)	1043 (59.4)	427 (42.5)	
Multiparous	1291 (46.8)	714 (40.6)	577 (57.5)	
Maternal education				<0.001
Compulsory secondary or below^a^	1662 (60.2)	949 (54.0)	713 (71.0)	
Upper secondary	486 (17.6)	323 (18.4)	163 (16.2)	
University degree or above	613 (22.2)	485 (27.6)	128 (12.8)	
Monthly family income (HKD)^b^				<0.001
<$15,000	692 (25.1)	269 (15.3)	423 (42.1)	
$15,000–$29,999	1159 (42.0)	757 (43.1)	402 (40.0)	
≥$30,000	910 (33.0)	731 (41.6)	179 (17.8)	
Marital status				0.107
Unmarried	161 (5.8)	112 (6.4)	49 (4.9)	
Married	2600 (94.2)	1645 (93.6)	955(95.1)	
Work full-time during pregnancy				<0.001
No	795 (28.8)	331 (18.8)	464 (46.2)	
Yes	1966 (71.2)	1426 (81.2)	540 (53.8)	
Work full-time post-partum				<0.001
No	1167 (42.3)	543 (30.9)	624 (62.2)	
Yes	1594 (57.7)	1214 (69.1)	380 (37.9)	
Maternal smoking				<0.001
No	2667 (96.6)	1679 (95.6)	988 (98.4)	
Yes	94 (3.4)	78 (4.4)	16 (1.6)	
Paternal smoking				0.085
No	1852 (67.1)	1199 (68.2)	653 (65.0)	
Yes	909 (32.9)	558 (31.8)	351 (35.0)	
Pre-pregnancy health problem				0.979
No	2525 (91.5)	1607 (91.5)	918 (91.4)	
Yes	236 (8.6)	150 (8.5)	86 (8.6)	
Pregnancy-related health problem				0.817
No	2387 (86.5)	1521 (86.6)	866 (86.3)	
Yes	374 (13.6)	236 (13.4)	138 (13.8)	
Delivery type				0.122
Spontaneous vaginal	1902 (68.9)	1187 (67.6)	715 (71.2)	
Assisted vaginal	139 (5.0)	96 (5.5)	43 (4.3)	
Planned cesarean	365 (13.2)	233 (13.3)	132 (13.2)	
Emergency cesarean	355 (12.9)	241 (13.7)	114 (11.4)	
Preterm birth				0.904
No	2546 (92.2)	1621 (92.3)	925 (92.1)	
Yes	215 (7.8)	136 (7.7)	79 (7.9)	
Infant LBW^c^				0.011
No	2540 (92.0)	1599 (91.0)	941 (93.7)	
Yes	221 (8.0)	158 (9.0)	63 (6.3)	

^a^Compulsory secondary education is to Form 3 or Grade 9.

^b^1 USD = 7.78 HKD

^c^*LBW* Low birth weight

Several characteristics were significantly associated with breastfeeding initiation in Hong Kong born and Mainland China born mothers (Table 2). In both groups, breastfeeding initiators were significantly more likely to be primiparas, have a university degree, work full-time during pregnancy and be non-smokers with non-smoking partners. In Hong Kong born participants, breastfeeding initiators were more likely to be older, married with a higher household income, and returning to work full-time postpartum. Hong Kong born participants who had a planned cesarean section were less likely to initiate breastfeeding and there was some evidence of lower breastfeeding in low birth weight infants, but the relationship was not statistically significant. In Mainland China born participants, breastfeeding initiation was not significantly associated with maternal age, household income, delivery status and returning to work postpartum but participants with pregnancy-related health problems were less likely to initiate breastfeeding. In both groups, breastfeeding initiation was not significantly associated with pre-pregnancy health problems or pre-term birth.

**Table 2:** Characteristics of breastfeeding initiators versus non-initiators by birthplace

Characteristic	Hong Kong			Mainland China			All		
	No	Yes		No	Yes		No	Yes	
	(*N* = 346)	(*N* = 1411)	*P*-value	(*N* = 190)	(*N* = 814)	*P-*value	(*N* = 536)	(*N* = 2225)	*P-*value
	N (%)	N (%)		N (%)	N (%)		N (%)	N (%)	
Maternal age (years)			.022			.158			.009
18–24	39 (24.5)	120 (75.5)		23 (21.7)	83 (78.3)		62 (23.4)	203 (76.6)	
25–29	97 (23.2)	322 (76.9)		55 (18.8)	238 (81.2)		152 (21.4)	560 (78.7)	
30–34	116 (16.6)	581 (83.4)		51 (15.4)	280 (84.6)		167 (16.3)	861 (83.8)	
≥35	94 (19.5)	388 (80.5)		61 (22.3)	213 (77.7)		155 (20.5)	601 (79.5)	
Parity			<.001			.002			<.001
Primiparous	156 (15.0)	887 (85.0)		62 (14.5)	365 (85.5)		218 (14.8)	1252 (85.2)	
Multiparous	190 (26.6)	524 (73.4)		128 (22.2)	449 (77.8)		328 (24.6)	973 (75.4)	
Maternal education			<.001			.039			<.001
Compulsory secondary or below^a^	257 (27.1)	692 (72.9)		148 (20.8)	565 (79.2)		405 (24.4)	1275 (75.6)	
Upper secondary	54 (16.7)	269 (83.3)		27 (16.6)	136 (83.4)		81 (16.7)	405 (83.3)	
University degree or above	35 (7.2)	450 (92.8)		15 (11.7)	113 (88.3)		50 (8.2)	563 (91.8)	
Monthly family income (HKD)^b^			<.001			.063			<.001
<$15,000	87 (32.3)	182 (67.7)		94 (22.2)	329 (77.8)		181 (26.2)	511 (73.8)	
$15,000–$29,999	169 (22.3)	588 (77.7)		69 (17.2)	333 (82.8)		238 (20.5)	921 (79.5)	
≥$30,000	90 (12.3)	641 (87.7)		27 (15.1)	152 (84.9)		117 (12.9)	793 (87.1)	
Length of time in Hong Kong						.219			
<5 years	--	--		45 (17.6)	211 (82.4)		--	--	
5 - <10 years	--	--		39 (16.2)	202 (83.8)		--	--	
≥10 years	--	--		104 (21.1)	388 (78.9)		--	--	
Marital status			<.001			.077			<.001
Unmarried	39 (34.8)	73 (65.2)		14 (28.6)	35 (71.4)		53 (32.9)	108 (67.1)	
Married	307 (18.7)	1338 (81.3)		176 (18.4)	779 (81.6)		483 (18.6)	2117 (81.4)	
Work full-time during pregnancy			<.001			.009			<.001
No	97 (29.3)	234 (70.7)		104 (22.4)	360 (77.6)		201 (25.3)	594 (74.7)	
Yes	249 (17.5)	1177 (82.5)		86 (15.9)	454 (84.1)		335 (17.0)	1631 (83.0)	
Work full-time post-partum			<.001			.139			.001
No	134 (24.7)	409 (75.3)		127 (20.4)	497 (79.7)		261 (22.4)	906 (77.6)	
Yes	212 (17.5)	1002 (82.5)		63 (16.6)	317 (83.4)		275 (17.3)	1319 (82.8)	
Maternal smoking			<.001			<.001			<.001
No	307 (18.3)	1372 (81.7)		179 (18.1)	809 (81.9)		486 (18.2)	2181 (81.8)	
Yes	39 (50.0)	39 (50.0)		11 (68.8)	5 (31.3)		50 (53.2)	44 (46.8)	
Paternal smoking			<.001			.001			<.001
No	191 (15.9)	1008 (84.1)		103 (15.8)	550 (84.2)		294 (15.9)	1558 (84.1)	
Yes	155 (27.8)	403 (72.2)		87 (24.8)	264 (75.2)		242 (26.6)	667 (73.4)	
Pre-pregnancy health problem			.330			.053			.707
No	321 (20.0)	1286 (80.0)		167 (18.2)	751 (81.8)		488 (19.3)	2037 (80.7)	
Yes	25 (16.7)	125 (83.3)		23 (26.7)	63 (73.3)		48 (20.3)	188 (79.7)	
Pregnancy-related health problem			.251			.011			.014
No	293 (19.3)	1228 (80.7)		153 (17.7)	713 (82.3)		446 (18.7)	1941 (81.3)	
Yes	53 (22.5)	183 (77.5)		37 (26.8)	101 (73.2)		90 (24.1)	284 (75.9)	
Delivery type			.044			.384			.095
Spontaneous vaginal	214 (18.0)	973 (82.0)		139 (19.4)	576 (80.6)		353 (18.6)	1549 (81.4)	
Assisted vaginal	21 (21.9)	75 (78.1)		8 (18.6)	35 (81.4)		29 (20.9)	110 (79.1)	
Planned cesarean	60 (25.8)	173 (74.3)		28 (21.2)	104 (78.8)		88 (24.1)	277 (75.9)	
Emergency cesarean	51 (21.2)	190 (78.8)		15 (13.2)	99 (86.8)		66 (18.6)	289 (81.4)	
Preterm birth			.105			.753			.138
No	312 (19.3)	1309 (80.8)		174 (18.8)	751 (81.2)		486 (19.1)	2060 (80.9)	
Yes	34 (25.0)	102 (75.0)		16 (20.3)	63 (79.8)		50 (23.3)	165 (76.7)	
Infant LBW^c^			.062			.720			.073
No	306 (19.1)	1293 (80.9)		177 (18.8)	764 (81.2)		483 (19.0)	2057 (81.0)	
Yes	40 (25.3)	118 (74.7)		13 (20.6)	50 (79.4)		53 (24.0)	168 (76.0)	

^a^Compulsory secondary education is to Form 3 or Grade 9

^b^1 USD = 7.78 HKD

^c^*LBW* Low birth weight

Table 3 presents the stratified and combined results of multivariable logistic regression analyses of predictors of breastfeeding initiation. In both the stratified and combined analyses, multiparity (OR 0.53, 95 % CI 0.43–0.66) and maternal smoking (OR 0.29, 95 % CI 0.18–0.45) were significantly associated with lower rates of breastfeeding initiation. In Hong Kong born participants, lower education levels and cesarean birth were associated with lower breastfeeding initiation. In Mainland China born participants, paternal smoking and having a pregnancy-related disease were also associated with lower rates of breastfeeding initiation. Family income was not clearly associated with breastfeeding initiation in either group of participants. Although in the stratified analysis, participants returning to work postpartum were less likely to initiate breastfeeding, the results were not statistically significant. In the combined analysis, however, participants who returned to work full-time were significantly less likely to initiate breastfeeding (OR 0.72, 95 % CI 0.54–0.95). Although preterm infants were not less likely to be breastfed, there is some indication that participants with low birth weight (LBW) infants were less like to initiate breastfeeding (OR 0.66, 95 % CI 0.43–1.02). However, the association did not reach the level of statistical significance. The results of the Hosmer–Lemeshow goodness-of-fit tests for the logistic regression models (*P* > .10) indicated that the models were a good fit for the data.

**Table 3:** Adjusted odds ratios (aOR) for breastfeeding initiation by birth place and for the total sample

	Hong Kong born		Mainland China born		All participants	
Characteristic	aOR^a^	(95 % CI)	aOR^a^	(95 % CI)	aOR^a^	(95 % CI)
Maternal age (years)						
18–24	1	--	1	--	1	--
25–29	0.71	(0.44, 1.16)	1.11	(0.61, 2.00)	0.86	(0.59, 1.25)
30–34	0.97	(0.59, 1.58)	1.47	(0.80, 2.71)	1.12	(0.77, 1.63)
≥35	0.90	(0.54, 1.51)	1.02	(0.55, 1.87)	0.97	(0.65, 1.43)
Parity						
Primiparous	1	--	1	--	1	--
Multiparous	0.46	(0.35, 0.61)	0.59	(0.40, 0.86)	0.53	(0.43, 0.66)
Maternal education						
Compulsory secondary or below^b^	0.29	(0.19, 0.45)	0.67	(0.35, 1.31)	0.36	(0.25, 0.52)
Upper secondary	0.42	(0.26, 0.67)	0.73	(0.36, 1.51)	0.49	(0.33, 0.72)
University degree or above	1		1		1	
Monthly family income (HKD)^c^						
<$15,000	1	--	1	--	1	--
$15,000–$29,999	1.10	(0.76, 1.58)	1.20	(0.82, 1.76)	1.06	(0.82, 1.36)
≥$30,000	1.42	(0.91, 2.23)	1.09	(0.61, 1.96)	1.20	(0.85, 1.68)
Marital status						
Unmarried	1	--	1	--	1	--
Married	1.24	(0.77, 2.00)	1.89	(0.93, 3.82)	1.46	(0.99, 2.14)
Work full-time during pregnancy						
No	1	--	1	--	1	--
Yes	1.42	(0.95, 2.12)	1.30	(0.83, 2.04)	1.29	(0.96, 1.74)
Work full-time post-partum						
No	1	--	1	--	1	--
Yes	0.74	(0.51, 1.06)	0.79	(0.49, 1.27)	0.72	(0.54, 0.95)
Maternal smoking						
No	1	--	1	--	1	--
Yes	0.36	(0.21, 0.61)	0.13	(0.04, 0.41)	0.29	(0.18, 0.45)
Paternal smoking						
No	1	--	1	--	1	--
Yes	0.80	(0.60, 1.05)	0.70	(0.49, 0.99)	0.75	(0.60, 0.92)
Pre-pregnancy health problem						
No	1	--	1	--	1	--
Yes	1.22	(0.76, 1.98)	0.65	(0.38, 1.10)	0.94	(0.66, 1.34)
Pregnancy-related problem						
No	1	--	1	--	1	--
Yes	0.85	(0.59, 1.21)	0.60	(0.38, 0.94)	0.73	(0.55, 0.96)
Delivery type						
Spontaneous vaginal	1	--	1	--	1	--
Assisted vaginal	0.60	(0.35, 1.03)	0.84	(0.37, 1.93)	0.65	(0.42, 1.03)
Planned cesarean	0.63	(0.44, 0.90)	0.94	(0.58, 1.53)	0.73	(0.55, 0.97)
Emergency cesarean	0.63	(0.43, 0.92)	1.52	(0.82, 2.82)	0.83	(0.61, 1.13)
Preterm birth						
No	1	--	1	--	1	--
Yes	0.96	(0.54, 1.70)	0.97	(0.47, 2.00)	1.00	(0.65, 1.55)
Infant LBW^d^						
No	1	--	1	--	1	--
Yes	0.64	(0.38, 1.09)	0.71	(0.32, 1.56)	0.66	(0.43, 1.02)

^a^adjusted for variables shown

^b^Compulsory secondary education is to Form 3 or Grade 9

^c^1 USD = 7.78 HKD

^d^*LBW* Low birth weight

## Discussion

In this study we examined breastfeeding initiation patterns among postpartum women born in Hong Kong and Mainland China and identified some common and disparate predictors of breastfeeding initiation. Overall, there was a high breastfeeding initiation rate among all participants, with equal rates in both groups. Multiparity and maternal and paternal smoking were risk factors for lower breastfeeding initiation in both groups. In Hong Kong mothers, as has been found in other studies [32, 33, 40, 41], breastfeeding initiation is strongly associated with higher maternal education. Hong Kong born participants with the lowest education level were 70 % less likely to initiate breastfeeding than participants in the highest education category. However, in contrast to studies in other countries [32, 33], higher family income was not significantly associated with breastfeeding initiation in either group. In Mainland Chinese women, breastfeeding initiation is less socially patterned with no significant associations between sociodemographic and socioeconomic indicators and initiation rates.

The breastfeeding initiation rate reported in this study is high and generally consistent with that reported by other parties. The annual Hong Kong breastfeeding survey reported that in 2010, 79.2 % of all mothers left the hospital breastfeeding [42]. However, the survey did find that in public hospitals, the same sample as our study, the breastfeeding initiation rate was only 71.3 %. Although breastfeeding initiation rates in Hong Kong are high relative to some other countries [43, 44], there is further room for improvement as many developed countries such as Australia, Canada, Denmark, Norway and Sweden all have breastfeeding initiation rates in excess of 90 % [3, 45, 46]. If breastfeeding initiation rates are to be improved beyond the current rate, targeted strategies are required. Thus, information gained from this study can be used to identify new mothers who are at risk for not initiating breastfeeding and provide them with additional evidence-based and cost-effective antenatal breastfeeding support. Furthermore, despite these high initiation rates, breastfeeding duration in Hong Kong remains short, with about one-half of all mothers who initiate breastfeeding stopping within the first three months [47]. Early breastfeeding cessation in Hong Kong has been attributed to sociocultural variables such as lack of family and community support for breastfeeding [26, 48–51], hospital practices that don’t adequately support breastfeeding [52–54], high workforce participation rates among childbearing women [29], and poor workplace accommodations for breastfeeding mothers [55]. Therefore, focus is also needed on how to sustain breastfeeding once it has been started and how best to provide support for breastfeeding mothers.

Consistent with previous studies [20–22, 40, 56], multiparity was negatively associated with breastfeeding initiation among all participants in this study. Why multiparous mothers are less likely to breastfeed is not clear. Multiparous mothers face greater time constraints in caring for multiple children and a second born child may be less likely to cause the same maternal anxiety and sense of novelty as compared with a firstborn [57]. In addition, when compared with primiparas, multiparous mothers may perceive less necessity to prove their maternal skills [58]. Other researchers have reported that mothers have more interaction with a first-born child than they have with subsequent children [57, 59]. These factors may contribute to lower breastfeeding initiation rates among multiparous mothers.

As has been found in other studies [21, 32, 40], we found that both maternal and paternal smoking were significantly associated with lower odds of breastfeeding. Although the overall rate of smoking in our population was low (3.3 %), especially when compared with rates reported in other studies [32, 40, 60], the impact was still substantial. When compared with non-smokers, participants who smoked were 60–80 % less likely to initiate breastfeeding. In addition, participants whose partners’ smoked were 20–30 % less likely to initiate breastfeeding. Because of public health messages encouraging smoking cessation during pregnancy and breastfeeding, new mothers who continue to smoke may believe that formula milk is healthier for their infant than breast milk from a smoking mother [40]. Other researchers have hypothesized that the relationship between maternal smoking and breastfeeding is confounded by socioeconomic status [21], whereby low rates of breastfeeding and high rates of smoking are more common in lower socioeconomic status individuals. Although we controlled for various socioeconomic indicators in our analysis and the relationship between smoking and breastfeeding initiation was still observed, we cannot rule out the possibility of residual confounding as smoking patterns are increasingly socioeconomically determined in most populations [61]. Given the consequences of maternal smoking on the fetus and on infants [62–64], pregnant women should be strongly encouraged to quit smoking. However, the benefits of breastfeeding outweigh the negative effects of exposure to nicotine through the breast milk and breastfeeding should still be promoted even in the absence of smoking cessation [65].

Birth by cesarean section was also a major barrier to breastfeeding initiation in Hong Kong born mothers. Although some studies have found no relationship between mode of delivery and breastfeeding initiation [66, 67], many have found that operative birth is a significant barrier to breastfeeding initiation [32, 68–71]. Previous studies, however, have rarely distinguished between the reasons for cesarean section (i.e., planned or emergency) and the impact on breastfeeding initiation. Overall, cesarean section rates were moderate in this study (26 %), especially in comparison to those reported in other countries [72–74], and are consistent with Hong Kong public hospital cesarean section rates reported elsewhere [75]. Furthermore, participants in this study undergoing a planned or scheduled cesarean section were doing so because it was medically indicated. Public hospitals in Hong Kong do not permit cesarean section upon maternal request, so all scheduled operative deliveries were deemed medically necessary. Thus, women who know that they will have a scheduled cesarean section may decide not to breastfeed because of anticipation of postoperative pain and discomfort. In addition, cesarean births and analgesia often increase maternal discomfort postpartum, cause separation of the mother and infant after birth, disrupt an infant’s natural breastfeeding reflexes, and thus increase problems with breastfeeding initiation [76, 77]. To allow mothers with cesarean sections adequate time to rest after surgery, infants are often given formula supplements before breastfeeding is initiated [53], further disrupting the initiation of breastfeeding. Therefore, antenatal breastfeeding education should target mothers known to be having a cesarean section and adequate postpartum support should be provided to all mothers who have a cesarean section to enable them to initiate breastfeeding as early as possible after birth.

Although preterm birth was not associated with breastfeeding, there was a strong, albeit statistically insignificant, association between LBW and breastfeeding initiation in the total sample. Because of the small number of infants born with LBW (*n* = 221) there was insufficient power to adequately assess this association. However, previous research in Hong Kong also found that LBW was a risk factor for failure to breastfeed [25] and studies in other countries have reported similar findings [78–80]. Although studies have repeatedly shown that human milk feeding of premature and LBW infants produces better health and developmental outcomes than infant formula [81–83], unfortunately many of these at risk newborns were never breastfed.

### Strengths and limitations

This study is one of the first to investigate the patterns and predictors of breastfeeding initiation in Hong Kong and Mainland China born mothers. The sample was large and population-based and captured a substantial proportion of eligible new mothers. Breastfeeding initiation status was extracted from the ward record book and then verified with the participants, thus increasing the accuracy of the outcome variable. Conversely, many of our other variables of interest relied on maternal self-report and response bias cannot be ruled out. However, given that the main focus of our original study was not breastfeeding [35], the possibility of social desirability bias was minimized. Furthermore, reported maternal smoking rates (3.3 %), an indicator that would be highly susceptible to social desirability bias, mirror the rates of female smoking (3.1 %) reported in population based surveys [84]. In addition, it is possible that breastfeeding decisions were influenced by variables that were not measured in our study such as the melamine tainted infant formula scandal in Mainland China [85]. Many experts agree, however, that the impact of this scandal on breastfeeding in Hong Kong and China was minimal [86] and it primarily served to create a large market for foreign infant formula brands in China [87, 88]. Finally, we do not have any information on mothers who refused to take part in this study and thus we do not know if they are similar or different from participants in ways that may affect the study findings.

## Conclusions

Results from this study show that breastfeeding initiation rates are high in Hong Kong born and Mainland China born mothers, with no significant differences between the two groups. However, there is potential to further increase breastfeeding initiation rates and data from this study has identified a number of predictors unique to each group that can help clinicians and public health professionals to further promote breastfeeding in Hong Kong. Given the current high breastfeeding initiation rates, evidence-based antenatal breastfeeding education and promotion programmes can be directed at sub-groups of pregnant women with lower initiation rates so that their efforts are more strategic and cost-effective.
